# Adeninium perchlorate

**DOI:** 10.1107/S1600536811001528

**Published:** 2011-01-15

**Authors:** Hoong-Kun Fun, Jia Hao Goh, Annada C. Maity, Shyamaprosad Goswami

**Affiliations:** a X-ray Crystallography Unit, School of Physics, Universiti Sains Malaysia, 11800 USM, Penang, Malaysia; b Department of Chemistry, Bengal Engineering and Science University, Shibpur, Howrah 711 103, India

## Abstract

In the title salt (systematic name: 6-amino-9*H*-purin-1-ium perchlorate), C_5_H_6_N_5_^+^·ClO_4_^−^, the adeninium cation is essentially planar, with a maximum deviation of 0.038 (1) Å. The whole of the perchlorate anion is disordered over two sets of sites with an occupancy ratio of 0.589 (13):0.411 (13). In the crystal, the adeninium cations are linked by pairs of N—H⋯N hydrogen bond into inversion dimers. The dimers and the anions are further inter­connected into a three-dimensional supra­molecular structure *via* inter­molecular N—H⋯O, C—H⋯O and C—H⋯N hydrogen bonds.

## Related literature

For general background to and applications of the title adeninium salt, see: Biradha *et al.* (2010 ▶); Goswami *et al.* (2007 ▶). For a closely related adeninium structure, see: Zeleňák *et al.* (2004 ▶). For the stability of the temperature controller used in the data collection, see: Cosier & Glazer (1986 ▶).
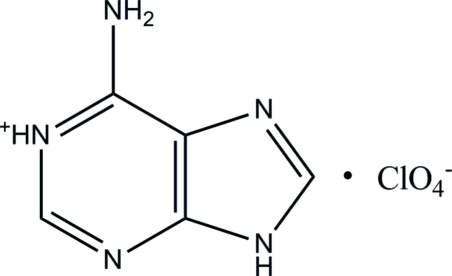


## Experimental

### 

#### Crystal data

C_5_H_6_N_5_^+^·ClO_4_^−^
*M* *_r_* = 235.60
Monoclinic, 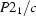

*a* = 8.7614 (2) Å
*b* = 4.8234 (1) Å
*c* = 21.0758 (4) Å
β = 112.070 (1)°
*V* = 825.39 (3) Å^3^
*Z* = 4
Mo *K*α radiation
μ = 0.47 mm^−1^
*T* = 105 K
0.29 × 0.28 × 0.20 mm

#### Data collection

Bruker SMART APEXII CCD area-detector diffractometer
Absorption correction: multi-scan (*SADABS*; Bruker, 2009 ▶) *T*_min_ = 0.878, *T*_max_ = 0.911
13078 measured reflections
3149 independent reflections
2538 reflections with *I* > 2σ(*I*)
*R*_int_ = 0.037

#### Refinement

*R*[*F* ^2^ > 2σ(*F* ^2^)] = 0.039
*wR*(*F* ^2^) = 0.101
*S* = 1.04
3149 reflections
200 parameters
10 restraints
All H-atom parameters refined
Δρ_max_ = 0.45 e Å^−3^
Δρ_min_ = −0.42 e Å^−3^

### 

Data collection: *APEX2* (Bruker, 2009 ▶); cell refinement: *SAINT* (Bruker, 2009 ▶); data reduction: *SAINT*; program(s) used to solve structure: *SHELXTL* (Sheldrick, 2008 ▶); program(s) used to refine structure: *SHELXTL*; molecular graphics: *SHELXTL*; software used to prepare material for publication: *SHELXTL* and *PLATON* (Spek, 2009 ▶).

## Supplementary Material

Crystal structure: contains datablocks global, I. DOI: 10.1107/S1600536811001528/is2652sup1.cif

Structure factors: contains datablocks I. DOI: 10.1107/S1600536811001528/is2652Isup2.hkl

Additional supplementary materials:  crystallographic information; 3D view; checkCIF report

## Figures and Tables

**Table 1:** Hydrogen-bond geometry (Å, °)

*D*—H⋯*A*	*D*—H	H⋯*A*	*D*⋯*A*	*D*—H⋯*A*
N1—H1*N*1⋯O3^i^	0.82 (2)	2.23 (2)	2.868 (7)	135.2 (17)
N3—H1*N*3⋯O4^ii^	0.79 (2)	2.07 (2)	2.818 (10)	158.2 (19)
N5—H1*N*5⋯N4^iii^	0.85 (2)	2.07 (2)	2.8938 (19)	164.3 (19)
N5—H2*N*5⋯O2^i^	0.85 (2)	2.28 (2)	3.100 (6)	162.0 (18)
C3—H3⋯N2^iv^	0.945 (19)	2.577 (19)	3.266 (2)	130.0 (15)
C3—H3⋯O1^v^	0.945 (19)	2.35 (2)	3.055 (6)	131.2 (16)
C5—H5⋯O4	0.94 (2)	2.45 (2)	3.174 (10)	133.6 (15)

Symmetry codes: (i) 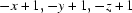; (ii) 
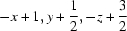
; (iii) 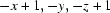; (iv) 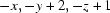; (v) 
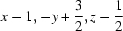
.

**Fig. 1.**
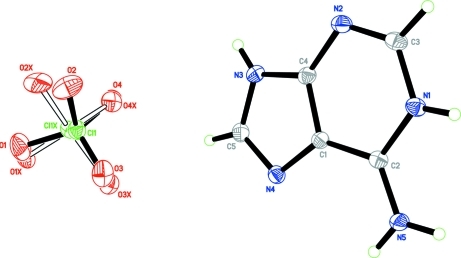
The molecular structure of the title salt, showing 50% probability displacement ellipsoids for non-H atoms and the atom-numbering scheme. Minor disordered component is shown as open bonds and labelled as suffix X.

**Fig. 2.**
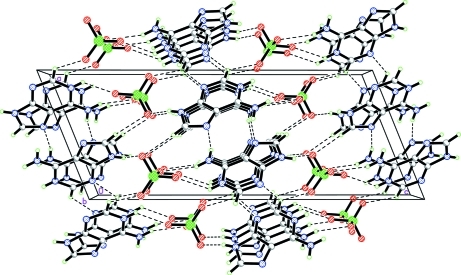
The crystal structure of the title salt, viewed along the b axis, showing a 3D supramolecular structure. Minor disordered component is omitted for clarity and intermolecular hydrogen bonds are shown as dashed lines.

## supplementary crystallographic information

### Comment

Adenine is a purine derivative nucleobase. Adenine is probably one of the most widely-used nucleobase in biochemistry (Biradha *et al.*, 2010). It is an integral part of DNA, RNA and ATP. As a nucleobase, adenine exhibits a tendency to self-associate with the help of Watson-Crick and Hoogsteen hydrogen bonds. We have recently reported the unique hydrogen bonding participation of H_5_O_2_^+^ bridging two hydrogen-bonded dimers of lumazine in its co-crystal with aqueous perchloric acid and the supramolecular assembly of protonated xanthine alkaloids in their perchlorate salts (Goswami *et al.*, 2007). In the present work, we report the crystal structure of adenine perchlorate.

The title salt comprises a protonated 6-amino-9*H*-purin-1-ium cation and a perchlorate anion (Fig. 1). The 6-amino-9*H*-purin-1-ium cation (C1–C5/N1–N5) is essentially planar, as indicated by the maximum deviation of 0.038 (1) Å at atom C1. The whole molecule of perchlorate anion (Cl/O1–O4) is disordered over two sites with refined occupancies of 0.589 (13) and 0.411 (13). All geometric parameters are consistent to a reported adeninium structure (Zeleňák *et al.*, 2004).

In the crystal packing, all hydrogen atoms take part in hydrogen bonding between the cation and anion. Intermolecular N1—H1N1···O3, N3—H1N3···O4, N5—H1N5···N4, N5—H2N5···O2, C3—H3···O1, C3—H3···N2 and C5—H5···O4 hydrogen bonds (Table 1) interconnect the ions into a three-dimensional supramolecular structure (Fig. 2).

### Experimental

Adenine (150 mg) was dissolved in perchloric acid (70 %, 1.0 ml) with gentle warming and the reaction mixture was kept at room temperature. After several days, colourless single crystals were separated, which were collected and dried.

### Refinement

All H atoms were located in a difference Fourier map, and allowed to refine freely with C—H = 0.942 (19)–0.95 (2) Å and N—H = 0.79 (2)–0.85 (2) Å. The whole molecule of perchlorate anion is disordered over two sites with a refined occupancy ratio of 0.589 (13):0.411 (13). Similarity restraints were applied for the perchlorate anion.

### Crystal data

**Table:**

C_5_H_6_N_5_^+^·ClO_4_^−^	*F*(000) = 480
*M**_r_* = 235.60	*D*_x_ = 1.896 Mg m^−^^3^
Monoclinic, *P*2_1_/*c*	Mo *K*α radiation, λ = 0.71073 Å
Hall symbol: -P 2ybc	Cell parameters from 4267 reflections
*a* = 8.7614 (2) Å	θ = 3.8–33.0°
*b* = 4.8234 (1) Å	µ = 0.47 mm^−^^1^
*c* = 21.0758 (4) Å	*T* = 105 K
β = 112.070 (1)°	Block, colourless
*V* = 825.39 (3) Å^3^	0.29 × 0.28 × 0.20 mm
*Z* = 4	

### Data collection

**Table:**

Bruker SMART APEXII CCD area-detector diffractometer	3149 independent reflections
Radiation source: fine-focus sealed tube	2538 reflections with *I* > 2σ(*I*)
graphite	*R*_int_ = 0.037
φ and ω scans	θ_max_ = 33.2°, θ_min_ = 2.5°
Absorption correction: multi-scan (*SADABS*; Bruker, 2009)	*h* = −13→13
*T*_min_ = 0.878, *T*_max_ = 0.911	*k* = −7→7
13078 measured reflections	*l* = −30→32

### Refinement

**Table:**

Refinement on *F*^2^	Primary atom site location: structure-invariant direct methods
Least-squares matrix: full	Secondary atom site location: difference Fourier map
*R*[*F*^2^ > 2σ(*F*^2^)] = 0.039	Hydrogen site location: inferred from neighbouring sites
*wR*(*F*^2^) = 0.101	All H-atom parameters refined
*S* = 1.04	*w* = 1/[σ^2^(*F*_o_^2^) + (0.043*P*)^2^ + 0.4566*P*] where *P* = (*F*_o_^2^ + 2*F*_c_^2^)/3
3149 reflections	(Δ/σ)_max_ < 0.001
200 parameters	Δρ_max_ = 0.45 e Å^−^^3^
10 restraints	Δρ_min_ = −0.42 e Å^−^^3^

### Special details

**Table:**

Experimental. The crystal was placed in the cold stream of an Oxford Cryosystems Cobra open-flow nitrogen cryostat (Cosier & Glazer, 1986) operating at 105.0 (1)K.
Geometry. All esds (except the esd in the dihedral angle between two l.s. planes) are estimated using the full covariance matrix. The cell esds are taken into account individually in the estimation of esds in distances, angles and torsion angles; correlations between esds in cell parameters are only used when they are defined by crystal symmetry. An approximate (isotropic) treatment of cell esds is used for estimating esds involving l.s. planes.
Refinement. Refinement of F^2^ against ALL reflections. The weighted R-factor wR and goodness of fit S are based on F^2^, conventional R-factors R are based on F, with F set to zero for negative F^2^. The threshold expression of F^2^ > 2sigma(F^2^) is used only for calculating R-factors(gt) etc. and is not relevant to the choice of reflections for refinement. R-factors based on F^2^ are statistically about twice as large as those based on F, and R- factors based on ALL data will be even larger.

### Fractional atomic coordinates and isotropic or equivalent isotropic displacement parameters (Å^2^)

**Table:**

	*x*	*y*	*z*	*U*_iso_*/*U*_eq_	Occ. (<1)
N1	0.14134 (14)	0.5810 (3)	0.41768 (6)	0.0152 (2)	
N2	0.14035 (14)	0.7456 (3)	0.52342 (6)	0.0169 (2)	
N3	0.33327 (15)	0.4802 (3)	0.61809 (6)	0.0177 (2)	
N4	0.43640 (13)	0.1928 (3)	0.56071 (6)	0.0153 (2)	
N5	0.30483 (15)	0.2191 (3)	0.40417 (6)	0.0162 (2)	
C1	0.32179 (15)	0.3695 (3)	0.51586 (7)	0.0137 (2)	
C2	0.25906 (15)	0.3820 (3)	0.44387 (7)	0.0134 (2)	
C3	0.08746 (17)	0.7514 (3)	0.45686 (7)	0.0167 (3)	
C4	0.25733 (16)	0.5486 (3)	0.55077 (7)	0.0148 (2)	
C5	0.43785 (16)	0.2674 (3)	0.62123 (7)	0.0174 (3)	
Cl1	0.7997 (9)	0.4578 (14)	0.7670 (3)	0.0170 (6)	0.589 (13)
O1	0.9452 (8)	0.4076 (15)	0.8255 (3)	0.0317 (11)	0.589 (13)
O2	0.7796 (6)	0.7499 (5)	0.7523 (3)	0.0294 (9)	0.589 (13)
O3	0.8120 (11)	0.3197 (11)	0.7080 (3)	0.0183 (8)	0.589 (13)
O4	0.6558 (10)	0.359 (2)	0.7785 (5)	0.020 (2)	0.589 (13)
Cl1X	0.8150 (12)	0.4603 (19)	0.7747 (5)	0.0155 (7)	0.411 (13)
O1X	0.9454 (11)	0.3192 (15)	0.8286 (5)	0.0216 (11)	0.411 (13)
O2X	0.8317 (7)	0.7552 (7)	0.7882 (5)	0.0285 (16)	0.411 (13)
O3X	0.8155 (16)	0.398 (2)	0.7091 (5)	0.0242 (16)	0.411 (13)
O4X	0.6614 (13)	0.362 (3)	0.7772 (7)	0.020 (3)	0.411 (13)
H1N1	0.098 (2)	0.598 (4)	0.3760 (10)	0.024*	
H1N3	0.322 (2)	0.554 (4)	0.6494 (11)	0.024*	
H1N5	0.382 (3)	0.103 (4)	0.4224 (10)	0.024*	
H2N5	0.259 (2)	0.223 (4)	0.3606 (10)	0.024*	
H3	0.005 (2)	0.880 (4)	0.4324 (10)	0.024*	
H5	0.501 (2)	0.182 (4)	0.6632 (10)	0.024*	

### Atomic displacement parameters (Å^2^)

**Table:**

	*U*^11^	*U*^22^	*U*^33^	*U*^12^	*U*^13^	*U*^23^
N1	0.0165 (5)	0.0140 (6)	0.0156 (5)	0.0027 (4)	0.0068 (4)	0.0033 (4)
N2	0.0161 (5)	0.0142 (6)	0.0212 (5)	0.0006 (5)	0.0079 (4)	−0.0016 (5)
N3	0.0183 (5)	0.0202 (6)	0.0150 (5)	0.0007 (5)	0.0068 (4)	−0.0037 (5)
N4	0.0143 (5)	0.0144 (5)	0.0158 (5)	0.0004 (4)	0.0042 (4)	0.0003 (4)
N5	0.0183 (5)	0.0157 (6)	0.0143 (5)	0.0049 (5)	0.0058 (4)	0.0006 (4)
C1	0.0140 (5)	0.0118 (6)	0.0151 (5)	−0.0008 (5)	0.0053 (4)	−0.0007 (5)
C2	0.0140 (5)	0.0108 (6)	0.0160 (5)	−0.0001 (5)	0.0065 (4)	0.0012 (5)
C3	0.0168 (5)	0.0121 (6)	0.0227 (6)	0.0013 (5)	0.0091 (5)	0.0017 (5)
C4	0.0149 (5)	0.0122 (6)	0.0176 (6)	−0.0020 (5)	0.0067 (4)	−0.0022 (5)
C5	0.0159 (5)	0.0190 (7)	0.0161 (6)	−0.0006 (5)	0.0047 (4)	−0.0008 (5)
Cl1	0.0164 (11)	0.0164 (7)	0.0169 (10)	−0.0001 (6)	0.0049 (8)	0.0010 (5)
O1	0.0236 (13)	0.050 (3)	0.0164 (13)	0.002 (2)	0.0015 (9)	0.003 (2)
O2	0.0461 (18)	0.0134 (10)	0.035 (2)	−0.0033 (10)	0.0222 (18)	−0.0004 (10)
O3	0.0203 (13)	0.020 (2)	0.0154 (11)	−0.0009 (18)	0.0073 (9)	0.0004 (14)
O4	0.016 (3)	0.026 (4)	0.024 (4)	−0.007 (2)	0.015 (3)	−0.001 (3)
Cl1X	0.0133 (13)	0.0132 (9)	0.021 (2)	−0.0005 (8)	0.0080 (15)	0.0022 (11)
O1X	0.0182 (16)	0.026 (3)	0.0145 (16)	0.007 (2)	−0.0008 (12)	0.005 (2)
O2X	0.033 (2)	0.0076 (12)	0.050 (4)	−0.0005 (13)	0.022 (2)	−0.0003 (16)
O3X	0.0252 (19)	0.037 (4)	0.0128 (17)	−0.007 (4)	0.0098 (13)	0.003 (3)
O4X	0.024 (5)	0.022 (6)	0.013 (4)	0.005 (4)	0.006 (3)	0.007 (4)

### Geometric parameters (Å, °)

**Table:**

N1—C2	1.3646 (18)	C1—C4	1.3855 (19)
N1—C3	1.3686 (18)	C1—C2	1.4075 (18)
N1—H1N1	0.82 (2)	C3—H3	0.95 (2)
N2—C3	1.3018 (18)	C5—H5	0.942 (19)
N2—C4	1.3571 (18)	Cl1—O1	1.423 (7)
N3—C5	1.361 (2)	Cl1—O2	1.439 (7)
N3—C4	1.3620 (18)	Cl1—O3	1.449 (7)
N3—H1N3	0.79 (2)	Cl1—O4	1.450 (7)
N4—C5	1.3208 (18)	Cl1X—O3X	1.417 (10)
N4—C1	1.3837 (17)	Cl1X—O1X	1.443 (9)
N5—C2	1.3153 (18)	Cl1X—O4X	1.446 (10)
N5—H1N5	0.85 (2)	Cl1X—O2X	1.447 (9)
N5—H2N5	0.85 (2)		
			
C2—N1—C3	123.92 (12)	N1—C3—H3	115.6 (12)
C2—N1—H1N1	118.7 (14)	N2—C4—N3	127.42 (13)
C3—N1—H1N1	117.4 (14)	N2—C4—C1	127.20 (12)
C3—N2—C4	112.27 (12)	N3—C4—C1	105.37 (12)
C5—N3—C4	106.86 (12)	N4—C5—N3	113.36 (12)
C5—N3—H1N3	126.2 (15)	N4—C5—H5	125.2 (12)
C4—N3—H1N3	126.9 (15)	N3—C5—H5	121.4 (12)
C5—N4—C1	103.49 (12)	O1—Cl1—O2	110.5 (5)
C2—N5—H1N5	119.2 (13)	O1—Cl1—O3	109.5 (6)
C2—N5—H2N5	122.7 (13)	O2—Cl1—O3	108.0 (5)
H1N5—N5—H2N5	118.0 (18)	O1—Cl1—O4	110.6 (6)
N4—C1—C4	110.93 (12)	O2—Cl1—O4	108.2 (6)
N4—C1—C2	130.69 (12)	O3—Cl1—O4	110.0 (7)
C4—C1—C2	118.28 (12)	O3X—Cl1X—O1X	112.0 (8)
N5—C2—N1	121.84 (12)	O3X—Cl1X—O4X	108.0 (10)
N5—C2—C1	124.83 (12)	O1X—Cl1X—O4X	106.8 (8)
N1—C2—C1	113.32 (12)	O3X—Cl1X—O2X	111.3 (7)
N2—C3—N1	125.00 (13)	O1X—Cl1X—O2X	108.5 (6)
N2—C3—H3	119.4 (12)	O4X—Cl1X—O2X	110.0 (9)
			
C5—N4—C1—C4	0.39 (15)	C3—N2—C4—N3	−177.16 (14)
C5—N4—C1—C2	−175.98 (14)	C3—N2—C4—C1	1.0 (2)
C3—N1—C2—N5	178.65 (13)	C5—N3—C4—N2	178.41 (14)
C3—N1—C2—C1	−0.51 (19)	C5—N3—C4—C1	−0.11 (15)
N4—C1—C2—N5	−1.6 (2)	N4—C1—C4—N2	−178.70 (13)
C4—C1—C2—N5	−177.75 (13)	C2—C1—C4—N2	−1.8 (2)
N4—C1—C2—N1	177.53 (13)	N4—C1—C4—N3	−0.17 (16)
C4—C1—C2—N1	1.38 (18)	C2—C1—C4—N3	176.70 (12)
C4—N2—C3—N1	0.0 (2)	C1—N4—C5—N3	−0.47 (16)
C2—N1—C3—N2	−0.2 (2)	C4—N3—C5—N4	0.38 (17)

### Hydrogen-bond geometry (Å, °)

**Table:**

*D*—H···*A*	*D*—H	H···*A*	*D*···*A*	*D*—H···*A*
N1—H1N1···O3^i^	0.82 (2)	2.23 (2)	2.868 (7)	135.2 (17)
N3—H1N3···O4^ii^	0.79 (2)	2.07 (2)	2.818 (10)	158.2 (19)
N5—H1N5···N4^iii^	0.85 (2)	2.07 (2)	2.8938 (19)	164.3 (19)
N5—H2N5···O2^i^	0.85 (2)	2.28 (2)	3.100 (6)	162.0 (18)
C3—H3···N2^iv^	0.945 (19)	2.577 (19)	3.266 (2)	130.0 (15)
C3—H3···O1^v^	0.945 (19)	2.35 (2)	3.055 (6)	131.2 (16)
C5—H5···O4	0.94 (2)	2.45 (2)	3.174 (10)	133.6 (15)

Symmetry codes: (i) −*x*+1, −*y*+1, −*z*+1; (ii) −*x*+1, *y*+1/2, −*z*+3/2; (iii) −*x*+1, −*y*, −*z*+1; (iv) −*x*, −*y*+2, −*z*+1; (v) *x*−1, −*y*+3/2, *z*−1/2.
